# *AmelOBP4*: an antenna-specific odor-binding protein gene required for olfactory behavior in the honey bee (*Apis mellifera*)

**DOI:** 10.1186/s12983-024-00554-y

**Published:** 2025-01-14

**Authors:** Fang Liu, Yu Lai, Lixian Wu, Qiang Li, Linyue Lei, Wei Yin, Yuan Zhang, Zachary Y. Huang, Hongxia Zhao

**Affiliations:** 1 Guangdong Key Laboratory of Animal Conservation and Resource Utilization, Guangdong Public Laboratory of Wild Animal Conservation and Utilization, Institute of Zoology, Guangdong Academy of Sciences, Guangzhou, 510260 People’s Republic of China; 2 The Core Facility, Zhejiang University School of Medicine, Zhejiang University, Hangzhou, 310058 China; 3 Yunnan Academy of Biodiversity, Southwest Forestry University, Kunming, 650224 Yunnan China; 4 Department of Entomology, Michigan State University, East Lansing, MI 48824 USA

**Keywords:** *AmelOBP4*, *Apis mellifera*, RNAi, Behaviour

## Abstract

**Background:**

Odorant binding proteins (OBPs) initiate the process of odorant perception. Numerous investigations have demonstrated that OBPs bind a broad variety of chemicals and are more likely to carry pheromones or odor molecules with high binding affinities. However, few studies have investigated its effects on insect behavior. Previously, we found that *AmelOBP4* has a significantly higher expression in the heads of foragers than that of nurses regardless of their ages, revealing its importance in foraging behaviour of the honey bee. RNA interference (RNAi) is the induction of sequence specific gene silencing by double-stranded RNA (dsRNA), it is a powerful tool that makes gene inactivation possible in organisms that were not amenable to genetic analysis before.

**Results:**

In this study, we found that *AmelOBP4* had high expression levels in the antennae of both nurses and foragers, and could be successfully inhibited by feeding double stranded RNA of *AmelOBP4* (ds*AmelOBP4*). Foragers with inhibited *AmelOBP4* showed significantly lower sugar responsiveness than control bees, and also significantly reduced EAG response to plant volatiles of nonanal, linalool and 1-Octen-3ol. On the other hand, nurses with inhibited *AmelOBP4* showed significantly reduced EAG response to brood pheromone of ethyl oleate, methyl linoleate, methyl palmitate and *β*-ocimene. Finally, the Y-tube choice assay showed nurses only exhibited a significantly reduced preference to ethyl oleate, but foragers exhibited significantly reduced preference to all these three plant volatiles.

**Conclusions:**

The findings of our study suggested that *AmelOBP4* plays an important role in the odorant binding process, especially in modulating olfactory behaviour in workers. Our results provide a foundation for exploring the olfactory mechanism of *Apis mellifera.*

**Supplementary Information:**

The online version contains supplementary material available at 10.1186/s12983-024-00554-y.

## Background

Olfaction is one of the oldest sensory systems, which includes peripheral and central subdivisions: the peripheral olfactory system screens and receives the odor molecules, and converts the chemical signals into electrical signals in neuronal cells; the central nervous system integrates and processes the electrical signals to induce the corresponding behavioral responses [1]. Odorant binding proteins (OBPs) are small and water-soluble proteins located in the sensillum lymph cavity of chemoreceptor organs [2], playing important roles in neuronal activation [3]. They send chemical signals to odorant receptors (ORs) that cause appropriate behavioral reactions in different insects, depending on the type of ligand [4, 5]. *Drosophila* flies with OBP lush mutant completely avoided 11-cis vaccenyl acetate (cVA), and were defective for aggregation behavior [6]. The mutants also showed a reduction in courtship and male–female discrimination in courtship behaviors [7]. OBPs have also been reported to be involved in the reception of certain oviposition attractants and the determination of reproductive sites by altering the sensitivity of the insect olfactory system [8, 9]. In addition, OBPs can control eating behavior by altering perception of host plant odorants or affecting sucrose intake in response to bitter chemicals [10, 11].

*Apis mellifera*, the Western honey bee, is a well-known model organism for studying a variety of fundamental scientific questions at the behavioral, neurological, and molecular levels [12]. All non-reproductive tasks in a colony are performed by the worker honey bees. The foragers (usually > 14d of age) travel outside to gather nectar, pollen, and water, while nurses (6 to 12d of age) perform the majority of work inside the hive, including feeding the larvae [13]. Olfaction plays an indispensable role as bees perform these tasks. Nurses can estimate the amount of brood in a colony by sensing the concentration of an odor produced by the brood [14]. Foragers have to evaluate the quality of the nectar or pollen and must then decide whether to exploit a food source or not [15], they also need to locate different sources (nectar, pollen, propolis or water), which is a task that heavily depends on odor recognition and learning [13, 44].

Based on its genome and bioinformatics analysis, 21 OBPs genes were identified in *Apis mellifera* [16], most were found in olfactory sensilla, and deliver the hydrophobic airborne compounds. For instance, *AmelOBP1* is able to recognize the queen pheromone [17], *AmelOBP2* and *AmelOBP13* are able to bind with various plant volatiles [18, 19], and *AmelOBP14* preferentially binds terpenoid molecules [20]. It has been confirmed that male-specific pheromone cVA can induce male-male aggression and promote sexual receptivity in female flies by regulation the transcript levels of Obp69a [21]. When bitter compounds are mixed with sucrose, OBP49a can bind with the bitter ligands, contributing to blocking of sugar taste detection in *Drosophila* [41, 42]. Knockdown of *LmigOBP4* significantly altered the behavioral traits of locusts [22]. BtabOBP4 can bind with β-ionone from the host plants during the oviposition behavior of *Bemisia tabaci* [23]. *AlinOBP4* protein binds strongly to the sex pheromone component E4O2H and some host plant volatiles, revealing its involvement in sex pheromone detection in male *A. lineolatus* [24]. However, it is still unknown whether OBPs are involved in the regulation of honey bee behavioral traits.

We previously identified the significantly higher expression of *AmelOBP4* in the heads of foragers than that of nurses regardless of their age [25], and speculated that it may be related to the division of labor in honey bees. In this study, we performed a large-scale search for OBPs in a wide variety of insects and analyzed their phylogenetic relationship, then characterized *AmelOBP4* expression patterns in different tissues of the honey bee by RT-qPCR. Moreover, RNAi was used to knockdown *AmelOBP4* to qualify the behavioral responses to sugar and the behavioral response to a battery of odorants. Our results enrich the understanding of function of OBP4 and provide clues for studying the roles of OBPs in other insects.

## Materials and methods

### Insects and body parts preparation

Bee colonies were maintained in Langstroth hives in Guangzhou city, Guangdong Academy of Sciences, China. To analyze gene expression in various adult body parts in different castes, antennae, head (excluding brain and antennae), brain, thorax, abdomen, legs and wings from “new bees” (freshly eclosed bees within 24 h), nurses (showing larvae-feeding behavior) and foragers (with pollen on hind legs) were dissected for RNA extraction. Each body part was collected from 15 workers in a colony. A total of 1000–1500 one-day-old bees were marked from a colony. Fifteen workers were collected at the following ages: 1, 5, 10, 15, 20, 25 and 30 d (with the day of emergence as day 1), and their antennae were dissected immediately and stored at – 80 °C for total RNA extraction. There were three replicates per time point.

### RT-qPCR analysis

Trizol protocol was used to extract total RNA from each body part of worker bees [28], the quality and quantity of which was detected using a NanoDrop (Thermo Fisher Scientific, Wilmington, DE, USA). Total RNA (1 μg per sample) was reverse transcribed with PrimeScript ™ RT Reagent Kit with gDNA Eraser (US Everbright, China). The reactions were performed in a TC PCR Thermocycle Instrument (BIOER) under the following conditions: 42 °C for 2 min, 37 °C for 15 min and 85 °C for 15 s. The q-PCR assays were performed in an ABI StepOnePlus™ Real-Time PCR system. Amplification was carried out in 20 μl reaction volume, containing 10 μl SYBR premix Ex Taq II (TaKaRa, Japan), 3 μl cDNA, 5.4 μl RNase free water, 0.8 μl of each of forward and reverse of the specific primer (10 μM, Table 1). PCR conditions were 95 °C for 30 s, 42 cycles of 95 °C for 5 s and 60 °C for 30 s, followed by the melting curve (60–95 °C). β*-actin* was used as the reference gene (Table 1). Relative mRNA expression was calculated using the 2^−△△Ct^ method [26].

**Table 1:** Primers used in this study for *AmelOBP4* double-stranded RNA synthesis and reverse-transcription quantitative polymerase chain reaction (RT-qPCR) analyses

gene	Application of primers	Primer sequence (5’–3’)
*AmelOBP4*	Primer for qPCR	F: GAGTCTGGAACTCGAGAACTAACACC R: CAACCATGCATTCGTCTTCGTCTG
*β-actin*		F: TGCCAACACTGTCCTTTCTG R: AGAATTGACCCACCAATCCA
ds*AmelOBP4*	Primer RNA interference	F: AGCAATTCTATGCTCGCAAAA R: CATCCTCCGTAAAGTCGTCG
		T7F:taatacgactcactatagggAGCAATTCTATGCTCGCAAAA T7R: taatacgactcactatagggCATCCTCCGTAAAGTCGTCG
ds*GFP*		F: taatacgactcactatagggGTGGAGAGGGTGAAGG R: taatacgactcactatagggGGGCAGATTGTGTGGAC

Underlined sequences indicate the T7 adaptor; F, forward primer; R, reverse primer

### dsRNA synthesis

ds*AmelOBP4* was used to knockdown the *AmelOBP4* expression, dsRNA of GFP (ds*GFP*) was used as the negative control. The dsRNAs were synthesized by using the T7 RiboMAX express RNAi system (Promega, WI, USA, P1700). Administration of dsRNA to nurses and foragers were conducted 2 h after the bees were collected from the colonies. Two micrograms of dsRNA contained in 10 μl 50% sugar solution was fed to individual bees manually. The dsRNA was delivered to the proboscis of bees by an Eppendorf pipette (Fig. S1). After each bee was fed double-stranded RNA, they were separated in a bee fixation tube (Fig. S1A) for 30 min, then pooled together into a rearing cages, fed with 50% sugar water. After 24 h, the antennae of these bees were dissected for total RNA extraction to examine the effects of RNAi.

### Sucrose responsiveness

Nurses and pollen foragers (50 bees per group) were captured in the morning from a typical colony, and restrained in the bee holding tube (Fig. S1). Half of them were fed with ds*AmelOBP4*, the rest with ds*GFP*. Proboscis extension reflex (PER) was used to test the sucrose responsiveness of bees 24 h after treatment [27]. Both antennae were touched with a droplet of increasing concentrations of sucrose: 0.1, 0.3, 1, 3, 10 and 30% (w: w) to test their sucrose responsiveness according to previous studies [28]. The proportions of individuals responding (dependent variable) to the concentration of sucrose solution offered (independent variable) were nonlinear. PER response (%) was analyzed after arcsine transformation which resulted in linear-response relationships used for analyses [29]. Sugar concentrations were treated as repeated measures.

### Electroantennogram (EAG) measurements

Based on previous receptor binding studies using recombinant protein OBP14 in both *Apis mellifera* [19] and *Apis cerana* [30], we selected ten plant volatiles (a-linolenic acid, citronellol, linalool, ethyl acetate, eugenol, methyl salicylate, myrcene, nonanal, 1-octen-3-ol and trans-caryophyllene) and four brood pheromone components (ethyl oleate, methyl linoleate, methyl palmitate, *β*-ocimene) for EAG test. The measurements of EAG were performed according to Zhao et al. [31] with a few modifications as follows: the whole antennae of adult bees were removed at the base, and both ends of antennae were carefully dissected, then immediately fixed to two-pronged electrode by Spectra 360 electrode gel (Parker Laboratories Inc., Fairfield, NJ, USA). The tested chemicals were dissolved in n-hexane to the final concentration of 300 μg/μl, and n-hexane was used as the blank control. Three technical repetitions and ten biological repetitions were carried out for each chemical.

### Y-tube olfactometer choices

A Y-tube olfactometer (stem 21 cm, arms 15 cm, at an angle of 60°, internal diameter of 8 mm) (Figure S2) was used for the bioassays. Incoming air created by an air pump system was filtered through activated charcoal and humidified with double distilled, deionized water. The filtered air was split between two holding glass wash bottles: one bottle served as a control and the other bottle held the test material. From each holding bottle, the air passed into the respective arms of the Y-tube. Airflow through the system was maintained at 300 ml/min by an inline flowmeter (Gilmont Instr., Barnant Co., Barrington, IL, United States). A daylight lamp of light-emitting diode (LED) was placed above the Y-tube for illumination. A 10 μl volume of the test chemicals (linalool, nonanal, 1-octen-3-ol, ethyl oleate, methyl linoleate, methyl palmitate, and β-ocimene) was diluted 100-fold, then added to a square filter paper (1 cm × 1 cm), which was placed in one bottle (Test). The same volume of n-hexane was dropped onto the same size paper in another bottle (Control). The whole Y-tube olfactometer setup was placed inside a fume hood, keeping the bees away from odors before test.

One adult bee (nurse or forager) was released at the end of the central tube, and its decision and the time it took to were recorded when it crawled the entire length of one arm. The bees were continually observed until they made a decision. Individuals who did not make a choice after 5 min were marked as not responding and were removed from the analysis. Each compound was evaluated on 45–60 bees from three colonies, each of which represented a replicate. All bees were starved for 2 h before being released. In all trials, the treatment arm was randomly assigned, and the Y-tube was rinsed in ethanol and air dried between replicates. Twelve bees were not used because they did not make a decision during the 5 min observation period, out of a total of 728 bees. The number of bees in the test tube was counted and the response ratio (number bees in test tube/number of bees in both tubes) was calculated [32].

### Homology modeling and molecular docking

Based on the *AcerOBP4* (KP717059) as a three-dimensional homologous mode, the third-order structure of AmelOBP4 protein was predicted by using the SWISS-MODEL online tool (https://swissmodel.expasy.org/). Structures of ligands were drawn with ChemBio Office 2010 software, and converted to pdb files with ChemBio3D ultra software. The molecular docking was carried out with Autodock tool 1.5.6. The docking models and hydrogen bonds were visualized in PyMol software.

### Data analyses

A mixed linear model (REML) in JMP 17.0 was used to analyze the *AmelOBP4* expression in different organs from three types of bee, if found significant, was followed by Tukey's honestly significant difference (HSD) test (based on Least Square Means) to compare the gene expression of *AmelOBP4* among the different organs. Colony was considered to be a fixed effect and type of tissue and bee type, and the interaction between the two, were treated as fixed effects. Each sample was considered as a repeated measure because the same sample provided different tissues. ANOVA was also used to analyze the data with PER as a dependent variable, where PER response (%) was analyzed after arcsine-square root transformation. Sugar concentrations were treated as repeated measures. Student’s t-test was used to analyze the gene expression of *AmelOBP4*, and EAG responses. Contingency table analysis was used to analyze the distribution of bees in the dsAmel*OBP4-*fed and ds*GFP-*fed bees. This method compares the distribution of the raw numbers of choices in both insect groups toward two odours (control vs test odour) and uses Chi-square statistic to determine if it is significantly different (P < 0.05) from a random distribution.

## Results

### Coding and amino acid sequences of *AmelOBP4*

**Fig. 1:**
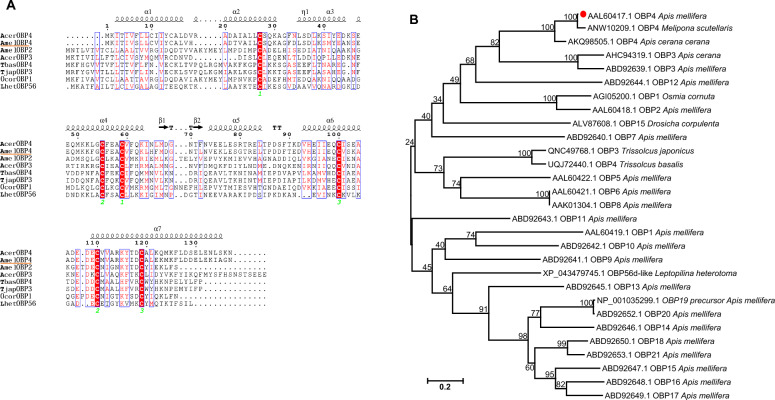
**A** Amino acid sequence alignment of AmelOBP4 with OBP4 from other species. TbasOBP4: OBP4 of *Trissolcus basalis;* OcorOBP1: OBP1 of *Osmia cornuta;* LhetOBP56d: OBP56d of *Leptopilina heterotoma*. Red box represents conserved amino acids domains including six highly conserved cysteines (labeled by green numbers below). The predicted secondary structures (e.g., α-helix) are shown above the corresponding sequences. **B** The phylogenetic tree of AmelOBPs family with other homologous proteins based on the method of Neighor-Joining (Bootstrap = 1000 times) using MEGA 6.0 software

We aligned the protein sequence of AmelOBP4 with other homologous sequences and its predicted secondary structure, and found that AmelOBP4 contained three pairs of disulfide bonds composed of six conserved cysteines (Fig. 1A). The *AmelOBP4* full-length ORF was 411 bp and the protein molecular weight was approximately 13.6 kDa. The phylogenic tree (Fig. 1B) showed that AmelOBP4 shared sequences with some homologous OBPs from diverse Hymenopterna species. The amino acid sequence of AmelOBP4 had high similarity to *Melipona scutellaris* OBP4 (96.32%) and *Apis cerana cerana* OBP4 (78.68%).

### Transcriptional profiling of *AmelOBP4* in various body parts and developmental ages

**Fig. 2:**
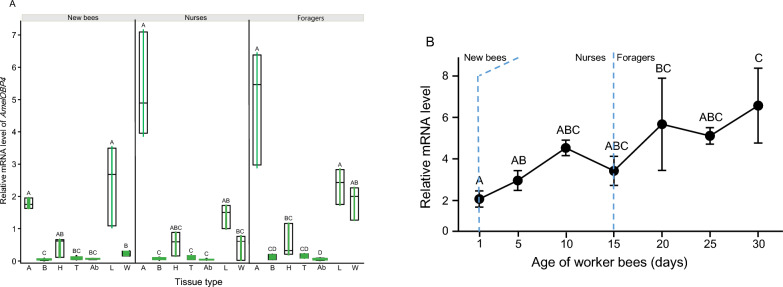
**A** The relative expression level of *AmelOBP4* in the antenna, brain, thorax, abdomen, leg, wing and head (without brain) from new bees, nurses and foragers. **B** *AmelOBP4* expression in antennae of worker bees in different ages (0–1: New bees; 5–15: nurses; > 15: foragers). Level of *AmelOBP4* mRNA were analyzed with REML analysis, followed by post-hoc Tukey test for multiple comparisons (n = 3 for each point). Different letters indicate significant differences. A: antennae; B: brain; H: head; T: thorax; Ab: abdomen; L: leg; W: wing

High throughput sequencing showed that *AmelOBP4* had significantly higher expression in the heads of foragers compared to the nurses regardless of their ages in a previous study [25]. RT-qPCR in this study confirmed these results (Fig. S3). Insect OBPs are not only primarily expressed in olfactory sensory cells, but also exist in non-olfactory tissues and involved in a variety of processes [33–35]. In order to investigate the function of *AmelOBP4* in honey bee, we determined its expression profiles in different tissues of the honeybee. As shown in Fig. 2, *AmelOBP4* showed significantly different expressions in three types of workers (F = 7.36, df = 2, 43, P < 0.05). There were highly significant differences in *AmelOBP4* expressions in different tissues (F = 44.79, df = 6, 43, P < 0.0001). The interactions between bee type and tissue type were not significant (F = 1.42, df = 12, 43, P > 0.2). In addition, we observed that expression of *AmelOBP4* in the antennae of worker bees varied significantly among different ages (F = 5.70, df = 6, 14; P < 0.01; Fig. 2B). The expression increased with age, reaching a maximum at 30 days of age.

### *AmelOBP4* decreases the sucrose responsiveness in foragers

**Fig. 3:**
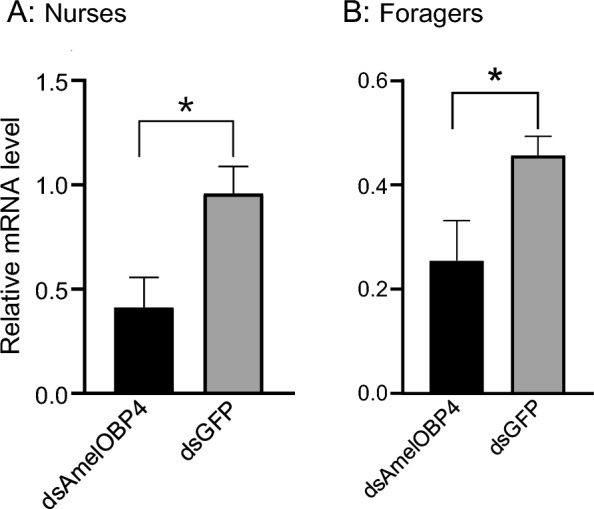
The mRNA level of *AmelOBP4* in the antennae of nurses (**A**) and foragers (**B**) after feeding with ds*AmelOBP4* or ds*GFP*. An independent t-test result is shown, data are presented as the mean ± SE (n = 3). An * indicates significant difference at P < 0.05 compared with the control group

To further investigate the possible function of *AmelOBP4* in the honey bee behavior, we tested the effect of *AmelOBP4* on PER (proboscis extension reflex) by feeding ds*AmelOBP4* to foragers and nurses. The expression of *AmelOBP4* in antennae from nurses (t = 2.79, P < 0.05) and foragers (t = 2.61; P < 0.05) were significantly suppressed at 24 h, a reduction of 44.2% and 57%, respectively, compared to the GFP control (Fig. 3). To determine whether ds*AmelOBP4* has an off-target effect, we tested the effect of ds*AmelOBP4* in foragers and measured the expression of the main *AmelOBP*s (*AmelOBP1*, *AmelOBP2*, *AmelOBP5*, *AmelOBP6*, *AmelOBP11*, *AmelOBP12*, *AmelOBP15*, and *AmelOBP4*)*.* The result shows that only* AmelOBP4* expression was significantly reduced (T = 3.23, P < 0.05, Fig. S4) and no significant reduction can be seen in any other *OBPs.* These data shows that our dsRNA was highly specific and only reduced the expression of *AmelOBP4*.

**Fig. 4:**
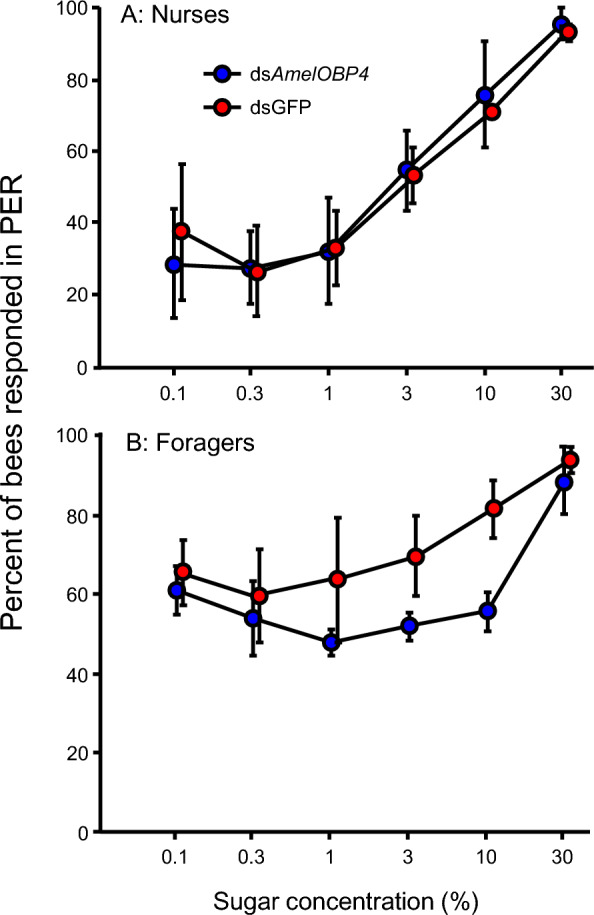
Mean score (% ± SE) of PER of foragers (**A**) and nurses (**B**) to various sugar concentrations after being treated with ds *AmelOBP4* or ds*GFP.* Responsiveness to sucrose was significantly (P < 0.01) decreased in ds*AmelOBP4* foragers, compared to the ds*GFP* control. There was no significantly different response (P > 0.05) to sucrose between nurses fed ds*AmelOBP4* and those fed ds*GFP*. Data from three colonies were analyzed after arsine transformation during ANOVA but presented here without transformation

PER response in foragers varied significantly with sugar concentrations (F = 36.94, df = 5, 12; P < 0.0001). The foragers fed with the ds*AmelOBP4* showed significantly lower PER response compared to the control bees fed with ds*GFP* (F = 142.04, df = 1, 5; P < 0.0001, Fig. 4B). There were no significant interactions between sucrose concentrations and the treatments (F = 1.62, df = 5, 10, P > 0.05). PER response in nurses varied significantly with sugar concentrations (F = 45.83, df = 5, 12; P < 0.001), while the PER response between nurses fed with the ds*AmelOBP4* and nurses fed with ds*GFP* showed no significant difference (F = 0.388, df = 1, 5; P > 0.05, Fig. 4A). There were no significant interactions between sucrose concentrations and the treatments (F = 0.583, df = 5, 10, P > 0.05). Moreover, nurses were significantly less responsive to sugar than foragers used in the above PER test (F = 32.65, P < 0.0001, Fig. S5).

### EAG response of nurses and foragers after knockdown of *AmelOBP4*

**Fig. 5:**
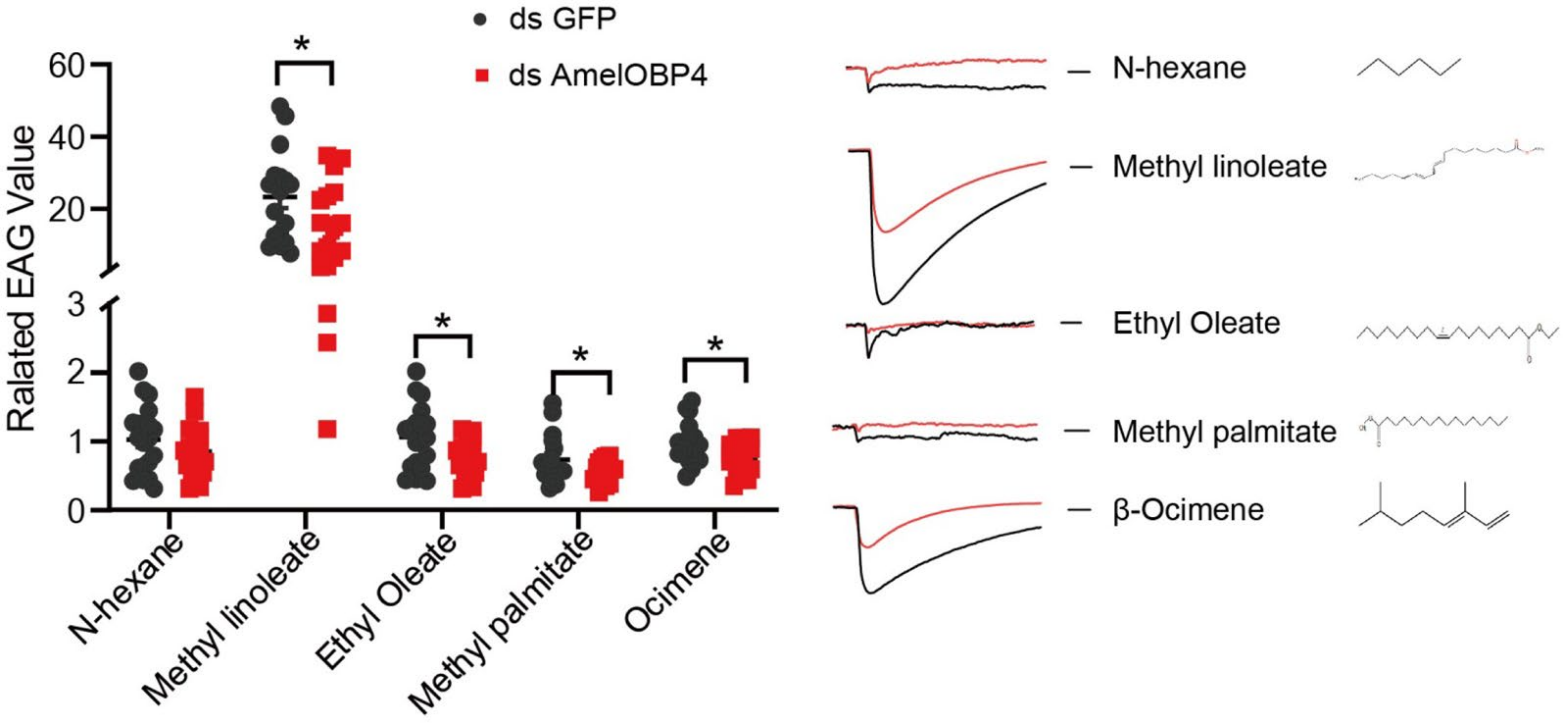
The EAG response of nurses to brood pheromone of methyl linoleate, ethyl oleate, methyl palmitate and β-ocimene, all data are means ± SE (*n* = 15). Asterisks represent a significant difference determined by ANOVA analysis (*p < 0.05)

**Fig. 6:**
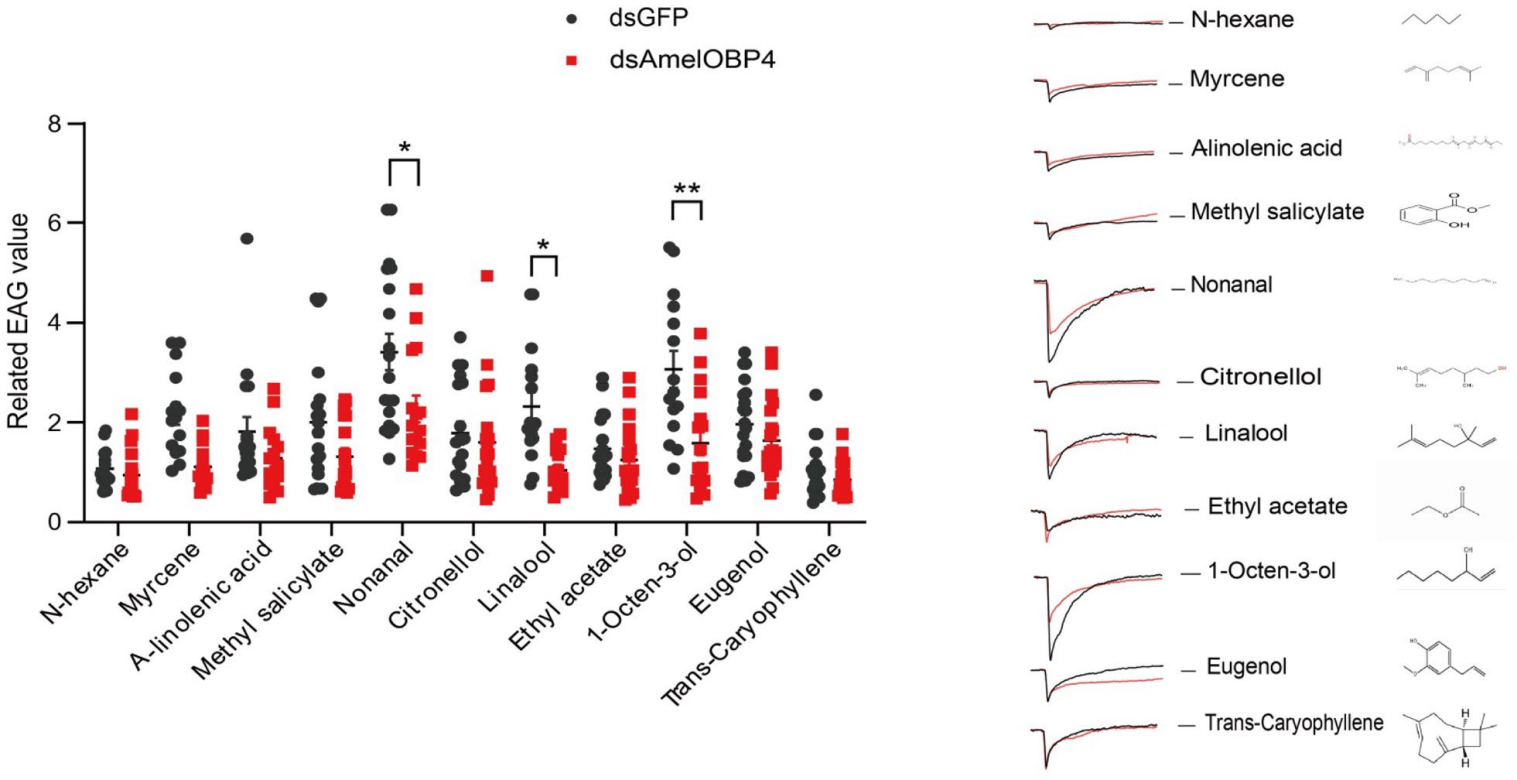
The EAG response of foragers to plant volatiles of n-hexane, myrcene, a-lindenic acid, methyl saliclate, nonanal, citronellol, linalool, ethyl acetate, 1-octen-3ol, eugenol and trans-caryophyllene, all data are means ± SE (*n* = 15). Asterisks represent a significant difference determined by ANOVA analysis (*p < 0.05; **p < 0.01)

Bees with high sucrose responsiveness are usually more sensitive to other stimulus modalities than bees with lower sucrose responsiveness [6]. To confirm this, an EAG assay after RNAi was conducted to assess the response of bees due to differences in *AmelOBP4* expressions. EAG values of all the tested compounds, except the solvent control (N-hexane) were significantly reduced in ds*AmelOBP4*-fed nurses compared to the control (Fig. 5, P < 0.05). In foragers, all the EAG values of the tested substance were somewhat reduced in the dsAmelOBP4 groups, in which the response to nonanal, linalool and 1-Octen-3ol were significantly reduced compared to the control (Fig. 6, p < 0.05).

### Y-tube of choice behavior of nurses and foragers

**Fig. 7:**
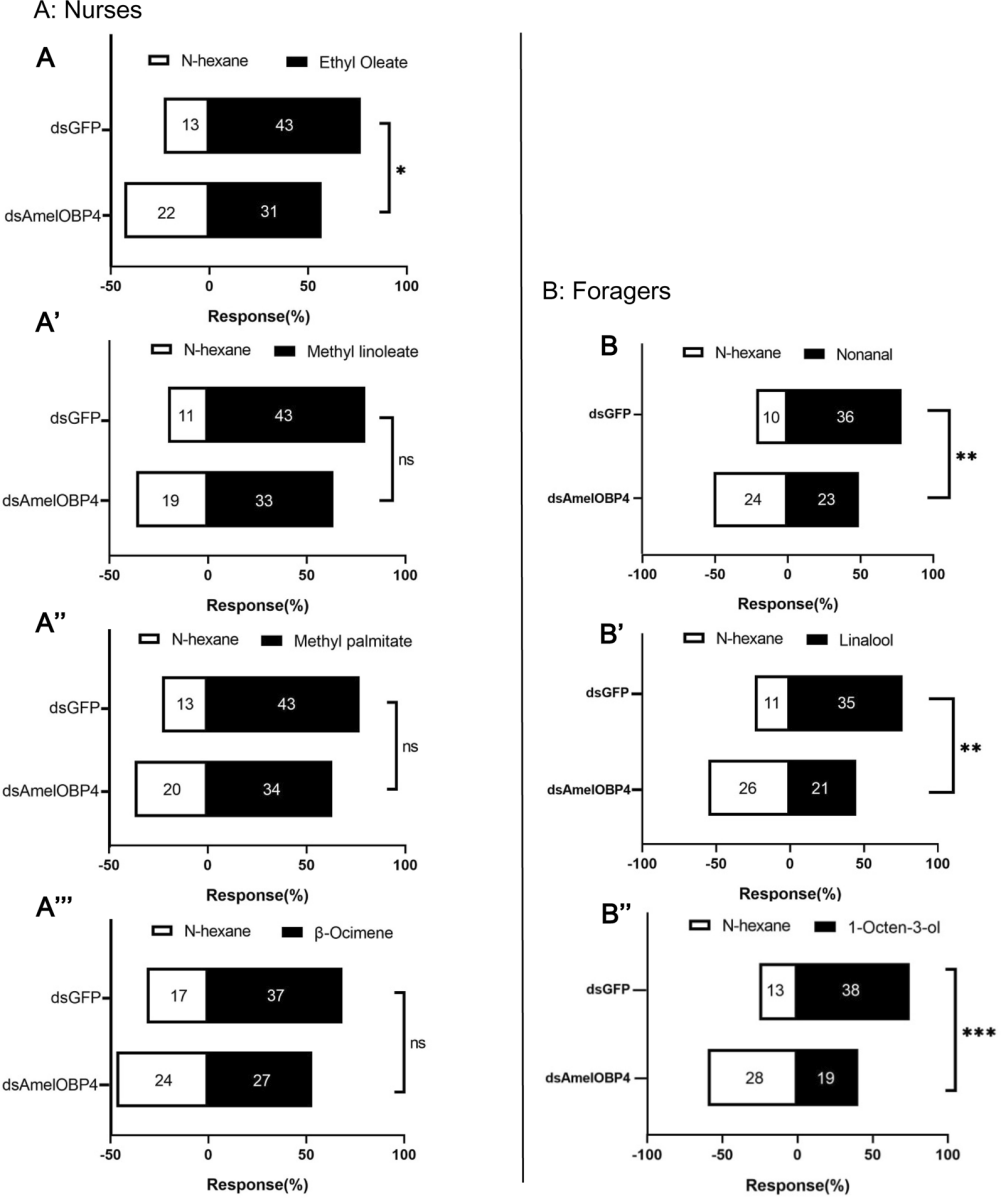
Quantification of nurses olfactory responses toward brood pheromones components: ethyl oleate (**A**), methyl linoleate (**A’**), methyl palmitate (**A’’**) and β-ocimene (**A’’’**) and forager olfactory responses toward plant volatiles of nonanal (**B**), linalool (**B’**) and 1-octen-3-ol (**B’’**). Statistical analysis was performed using a Chi square test. **: p < 0.001; *: p < 0.05, ns: p > 0.05

Based on the EAG response to brood pheromones and plant volatiles in nurses and foragers, we speculate that nurses and foragers must have behavioral response to these compounds. Indeed, a significantly lower attraction to ethyl oleate (χ^2^ = 4.21, *P* = 0.04, Fig. 7A) was found in nurses fed with ds*AmelOBP4* compared with the control (ds*GFP*). While they showed no significant changes in response to methyl palmitate (χ^2^ = 2.514, *P* = 0.1138, Fig. 7A''), methyl linoleate (χ^2^ = 3.441, *P* = 0.0647, Fig. 7A') and β-ocimene (χ^2^ = 2.684, *P* = 0.1020, Fig. 7A'''). Extremely significantly lower attraction to 1-octen-3ol (χ^2^ = 11.91, *P* = 0.0006, Fig. 7B''), linalool (χ^2^ = 9.786, *P* = 0.002, Fig. 7B') and nonanal (χ^2^ = 8.817, *P* = 0.0033, Fig. 7B) were found in foragers fed with ds*AmelOBP4*.

### Molecular docking

**Fig. 8:**
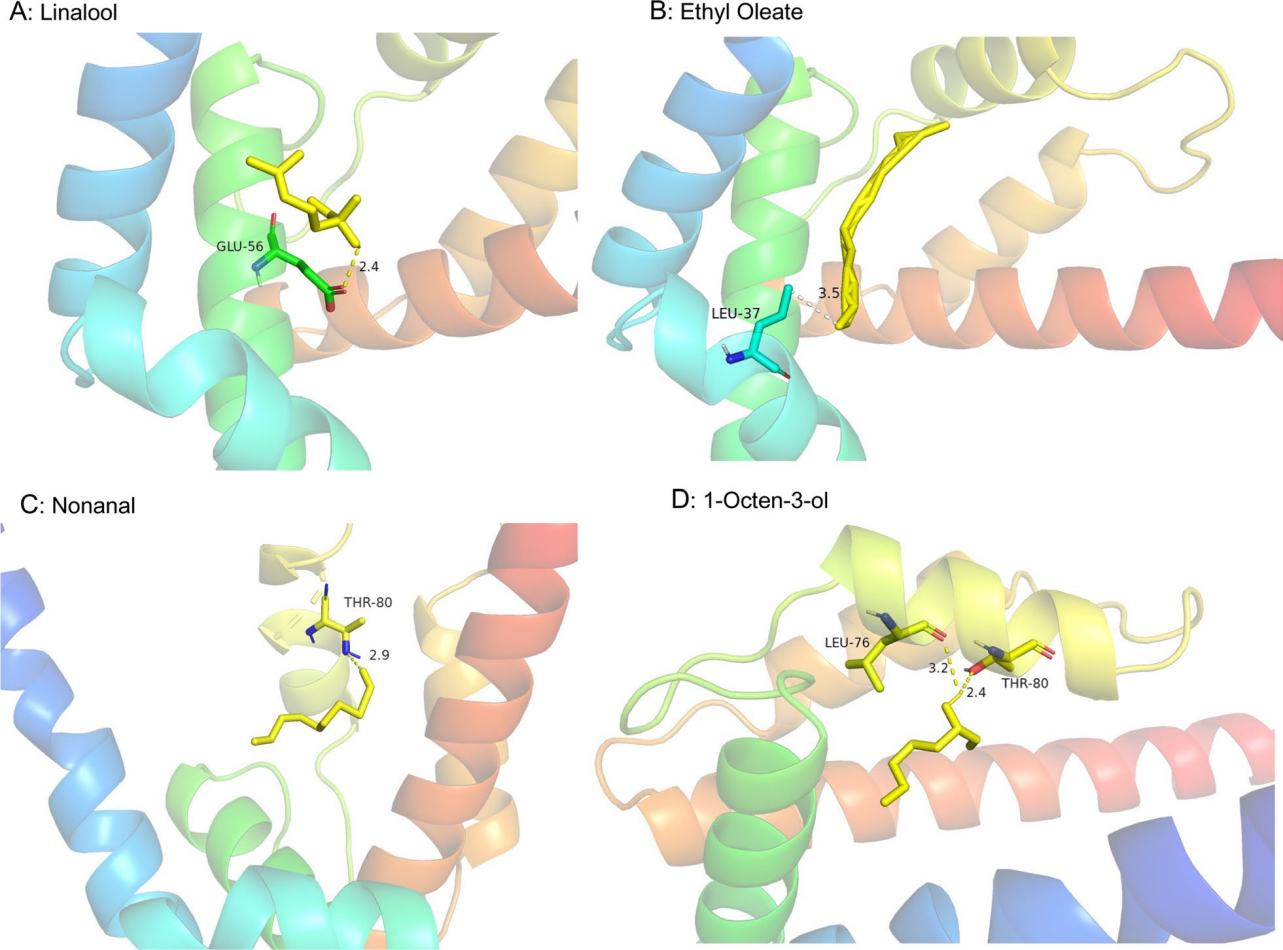
Hydrogen bonds formed between ethyl oleate, linalool, nonanal, 1-Octen-3-ol and AmelOBP4 as predicted by Auto dock tool. The substrates ethyl oleate, linalool, nonanal and 1-octen-3-ol are shown as water blue, potential hydrogen bonds are indicated by blue dotted lines, ligands are shown in yellow

Based on the odorant binding protein 4 from *Apis cerana cerana* as a three-dimensional homologous model, a homology model of the AmelOBP4 was predicted, the similarity of amino acid sequences between AmelOBP4 and the AcerOBP4 was 78.68%, and GMQE (Global Model Quality Estimation) was 0.85 (Fig. S6). Ethyl oleate, linalool, nonanal, and 1-octen-3-ol showed interactions with AmelOBP4, the hydrogen bonds were formed between ethyl oleate, linalool, nonanal, 1-octen-3-ol and the amino acids of AmelOBP4 (Table 2, Fig. 8), Leu37, Glu56, Thr80 and Leu76 were involved in the formation of these hydrogen bonds.

**Table 2:** Docking parameters between ethyl linolenate, ethyl palmitate, methyl linoleate, ethyl oleate, methyl palmitate and the amino acids of AmelOBP4

PubChem IDs	Ligands	Binding energy (kca/mol)	Residues interacting with H-Bonding
111-62-6	Ethyl Oleate	− 4.8	Leu37
78-70-6	Linalool	− 4.4	Glu56
3391-86-4	1-Octen-3-ol	− 4.2	Thr80; Leu76
124-19-6	Nonanal	− 3.6	Thr80

## Discussion

Numerous studies have confirmed that OBPs expressed specifically in the antennae and regulate the function of insect olfaction [36], while the ones expressed in other organs are involved in the non-chemosensory processes [22, 37, 38]. In this study, *AmelOBP4* was confirmed to have a higher expression in the antennae and legs compared to other body parts in both nurses and foragers, suggesting its function in recognizing general odorants of bee colonies [14, 15]. This result is similar to our previous finding of *AcerOBP4* [39]. Additionally, *AmelOBP4* steadily increases with age, it changes early in the bee's life but less so later, and remains stable even when bees switch from nurses to foragers, revealing that *AmelOBP4* may have no effect on the division of labor of honey bee, but may play other roles in their behaviours.

PER offers a behavioral readout for perceptual encoding of tastants since it is triggered when insects' gustatory receptors come into touch with appetitive stimuli [40]. Individual responsiveness to sucrose is measured by applying a series of sucrose concentrations to the antennae of a bee in this study. Bees with high sucrose responsiveness are more responsive to gustatory and olfactory than bees with low sucrose responsiveness [15]. Here, knock down of *AmelOBP4* attenuated the responsiveness of foragers to sucrose (Fig. 4), and reduced their sensitivity to plant volatiles (Fig. 6). These suggest that *AmelOBP4* plays an important role in the olfactory behavior of honey bee. Researchers also have capitalized on PER by using a Pavlovian conditioning technique to evaluate learning and memory [41, 42]. Sucrose responsiveness is strongly correlated with tactile and olfactory learning performance in foragers [43, 44]. Learning performance was significantly better when sucrose responsiveness was high than when it was low [45–47]. In this study, suppressing of *AmelOBP4* attenuated the responsiveness of foragers may also have effect on their learning performance. However, it needs to be further validated. Unexpectedly, nurses also have high expression of *AmelOBP4* in antennae, while silencing of *AmelOBP4* has no effect on their sugar response. It may have something to do with the fact that nurse bees themselves are less sensitive to sugar than foragers (Fig. S6). An alternative explanation is that *AmelOBP4* is not simply binding odorants on the antenna, but has additional functions.

Insect OBPs silencing or knockout can lead to their abnormal behavioral responses to different odorants. For example, *Bactrocera dorsalis* behavioral response to methyl eugenol was considerably diminished following the CRISPR/Cas9 knockout of *BdorOBP69a*, *BdorOBP56f-2*, or *BdorOBP13* [48–50]. The behavioural response of *Diaphorian citri* to host plant volatiles was reduced after *DcitOBP7* was partially silenced by RNAi [51]. In this study, we detected the decreased preference to compounds in ds*AmelOBP4* nurses and ds*AmelOBP4* foragers. Interestingly, the antennae from nurse bee showed decreased EAG response to β-ocimene after knockdown of *AmelOBP4*, but RNAi did not affect these nurses’ choice to β-ocimene (Fig. 7A). β-ocimene inhibits worker ovary development and accelerates the behavioral transition from nursing to foraging in *Apis mellifera* [52].Worker bees could potentially assess the concentration of β-ocimene in a colony—possibly as an indication of the amount of young brood in the colony and adjust their behaviors accordingly [53]. Taken together, we may speculate that *AmelOBP4* does not influence behavioral shifts in bees, but may have effect on bee feeding behavior.

Molecular docking can reveal amino acids that mediate ligand binding [2, 54, 55]. We identified Leu37, Glu56, Thr80 and Leu76 as potential regulators of ligand binding. Nonanal and 1-octen-3-ol possessed the same hydrogen bond (Thr80) interaction with AmelOBP4. The binding energy of Leu37 between ethyl oleate and AmelOBP4 was the strongest, suggesting that ethyl oleate is one of the odorants specifically recognized by AmelOBP4. This needs to be verified in future studies.

## Conclusion

In this study, we showed that *AmelOBP4* is highly expressed in the antennae of nurses and foragers. Feeding *dsAmelOBP4* can significantly reduce the expression of *AmelOBP4* in the antennae, which decreases the sucrose responsiveness in foragers, but not on nurses. Nurses with knockdown of *AmelOBP4* showed significantly reduced EAG response to brood pheromone components ethyl oleate, methyl palmitate, methyl linoleate, and β-ocimene. Foragers with knockdown of *AmelOBP4* showed significantly reduced EAG response to plant volatiles nonanal, linalool and 1-octen-3ol. Moreover, nurses with silenced *AmelOBP4* showed significantly reduced preference to ethyl oleate, and foragers showed remarkable significantly reduced preference to nonanal, linalool and 1-octen-3ol after knockdown of *AmelOBP4*. Our results provide credible evidence that suppressing *AmelOBP4* significantly weakens the olfactory reactions of *Apis mellifera*.

## Supplementary Information

Supplementary material 1.

## Data Availability

Data generated and/or analyzed during the current study are available from the corresponding authors on reasonable request.
