# Supplementary material for: AmelOBP4: an antenna-specific odor-binding protein gene required for olfactory behavior in the honey bee (Apis mellifera)
Source: Front Zool. 2025 Jan 14;22:2. doi: 10.1186/s12983-024-00554-y (PMC11731170; doi:10.1186/s12983-024-00554-y)
Supplement: Supplementary file 1 — Supplementary material 1. [file 12983_2024_554_MOESM1_ESM.pptx]

## Slide 1
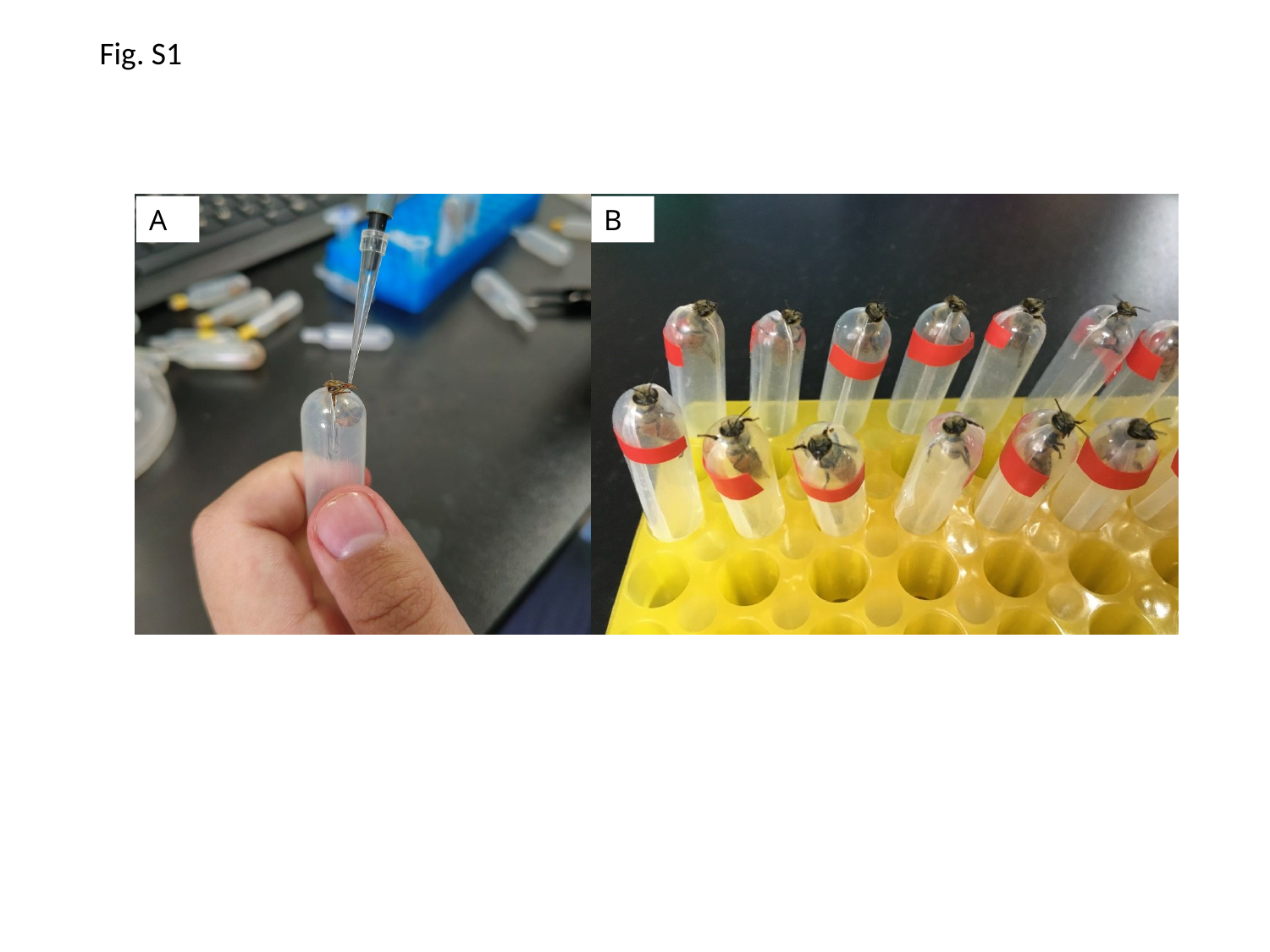

Fig. S1
A
B

## Slide 2
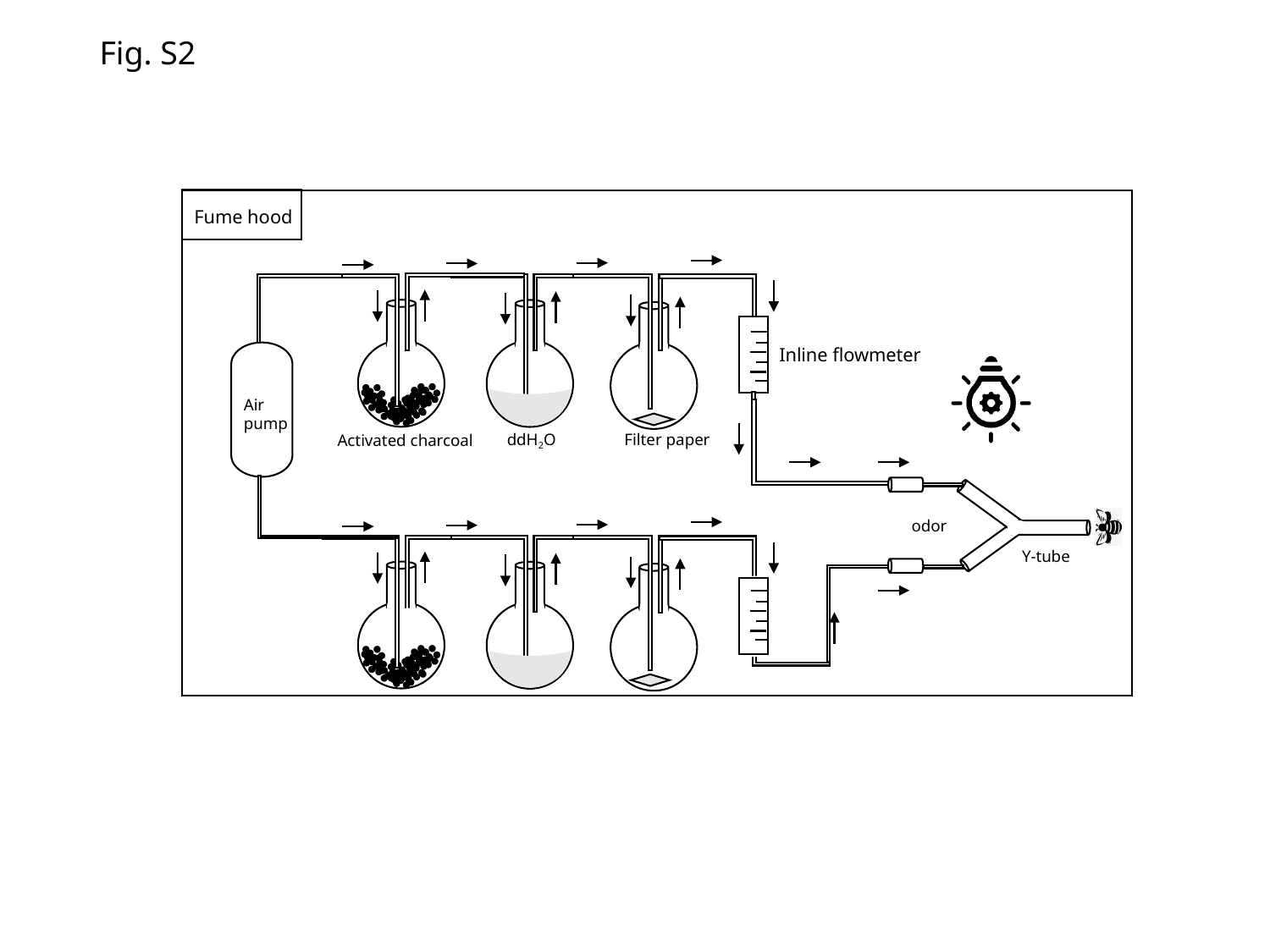

Fig. S2
Fume hood
Inline flowmeter
Air
pump
ddH2O
Filter paper
Activated charcoal
odor
Y-tube

## Slide 3
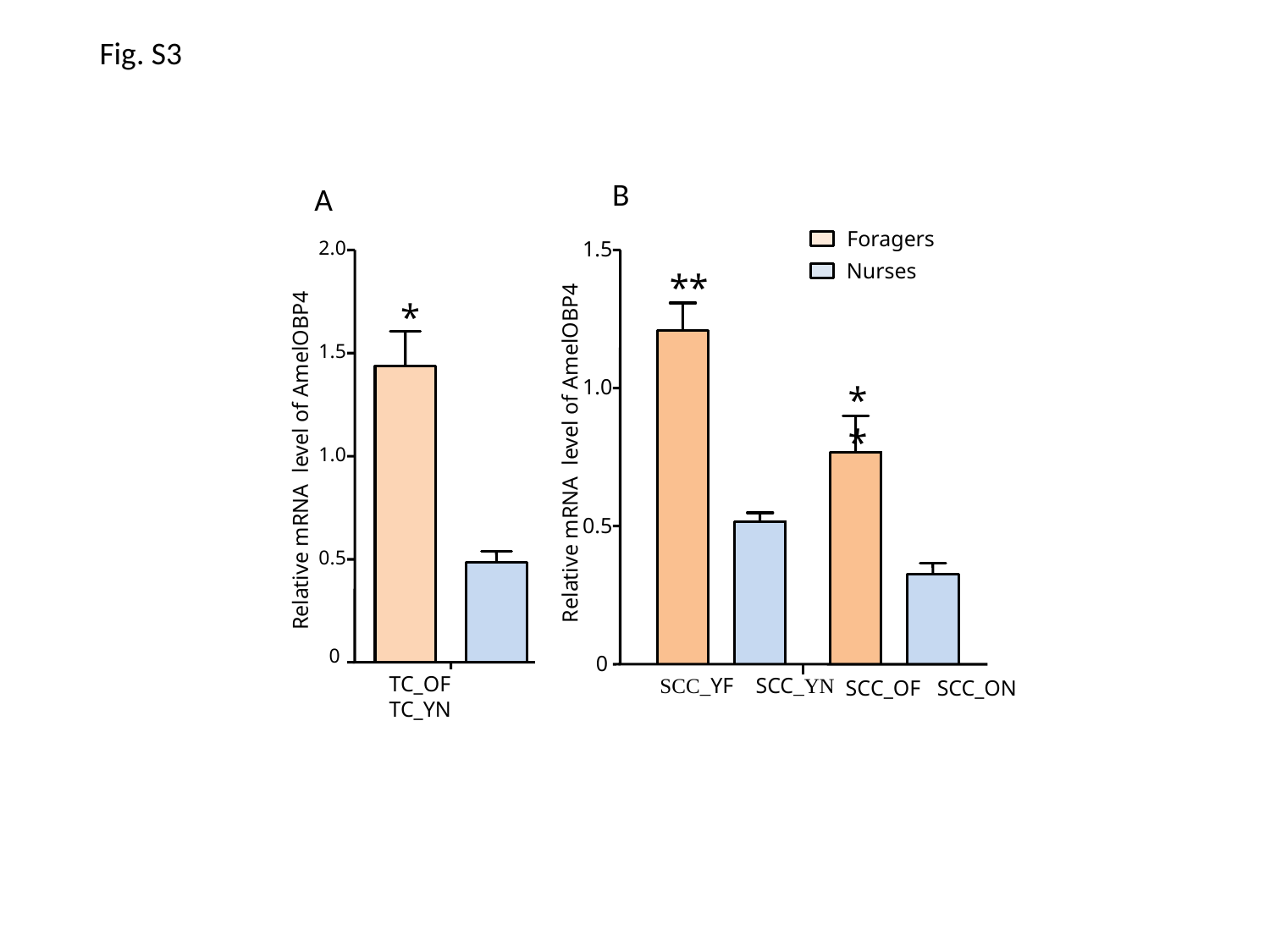

Fig. S3
B
A
Foragers
2.0
1.5
Relative mRNA level of AmelOBP4
1.0
0.5
0
1.5
1.0
Relative mRNA level of AmelOBP4
0.5
0
SCC_YF SCC_YN
SCC_OF SCC_ON
Nurses
**
*
**
TC_OF TC_YN

## Slide 4
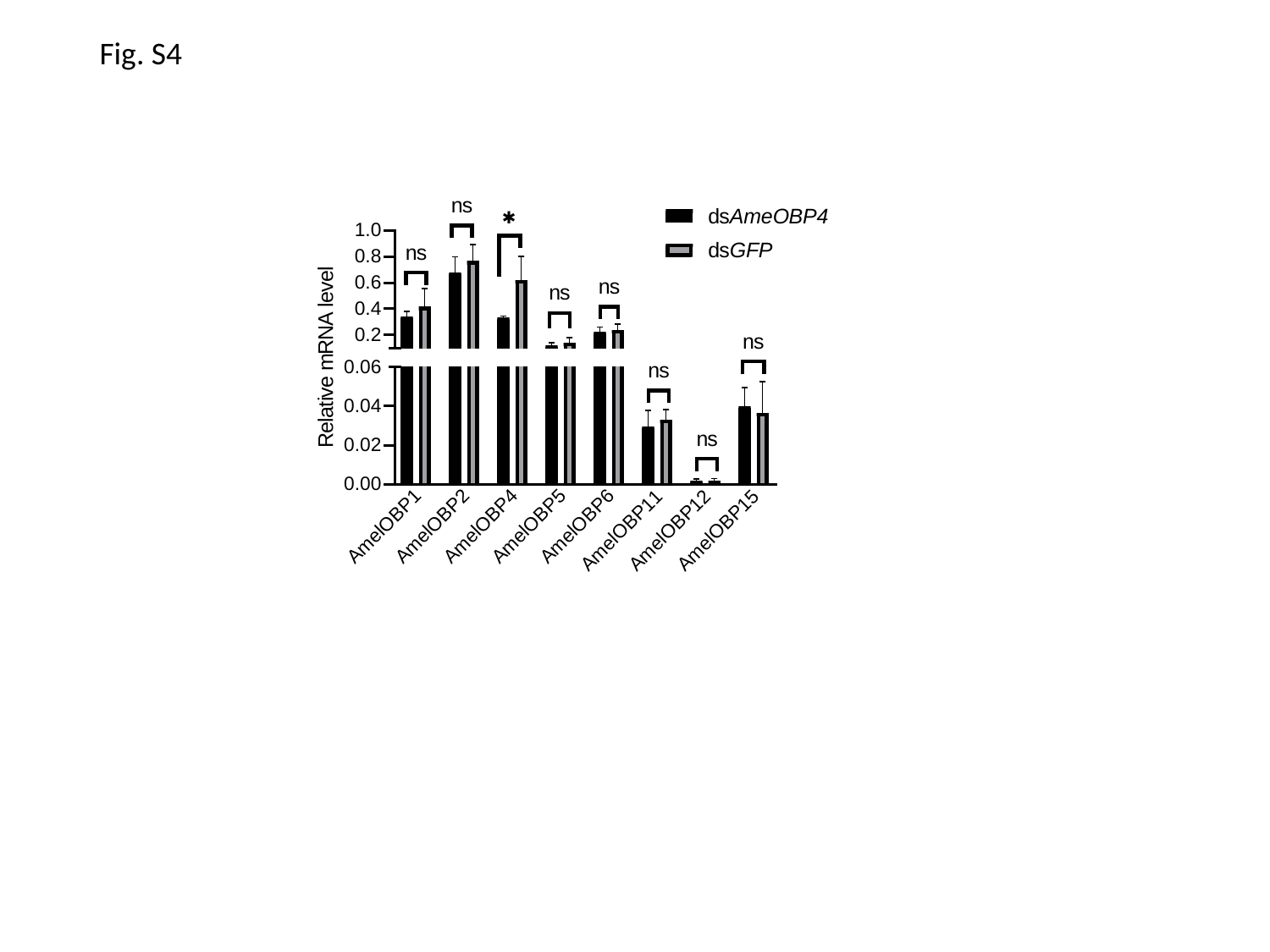

Fig. S4

## Slide 5
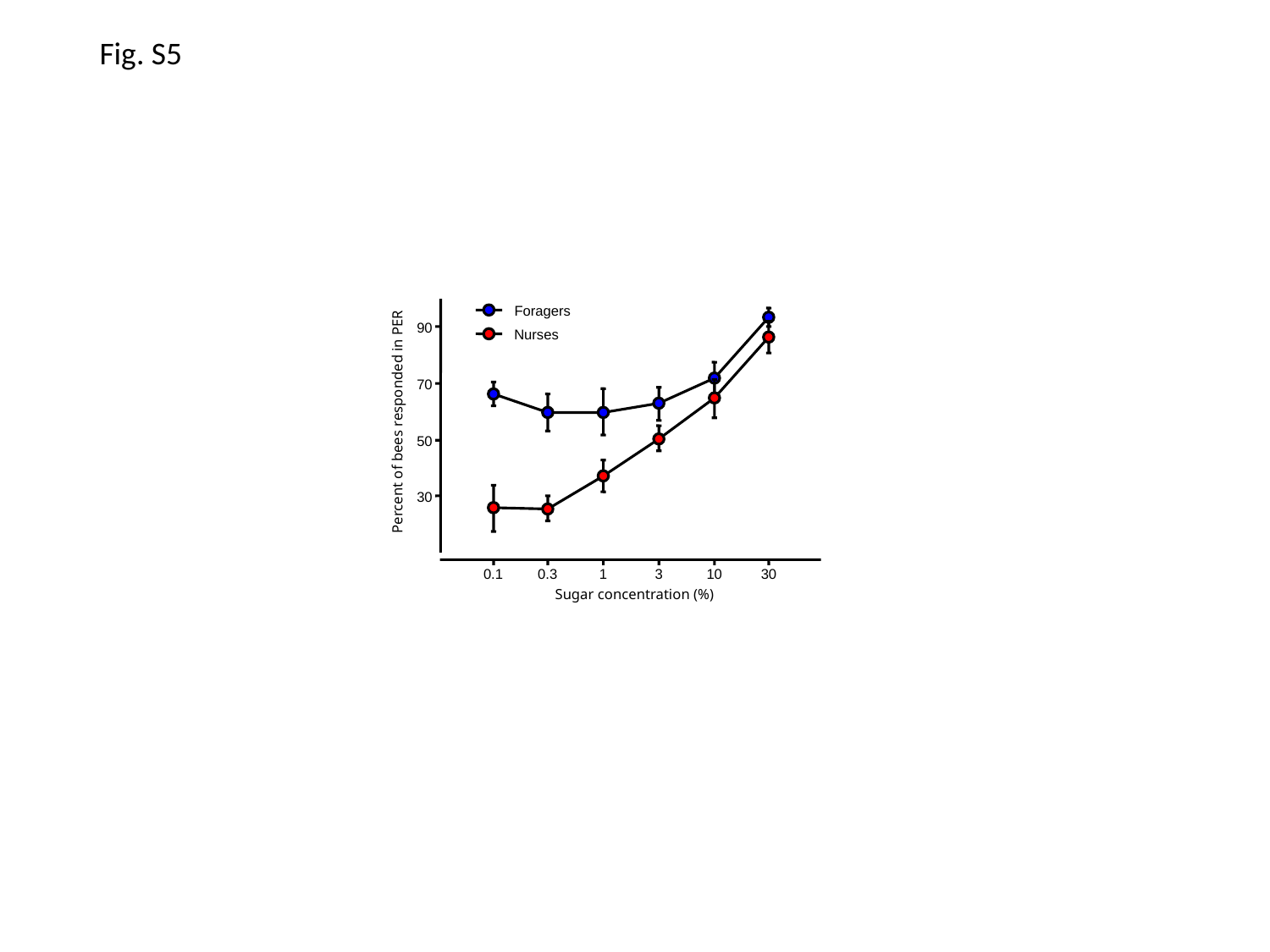

Fig. S5
Foragers
90
Nurses
70
Percent of bees responded in PER
50
30
0.1
0.3
1
3
10
30
Sugar concentration (%)

## Slide 6
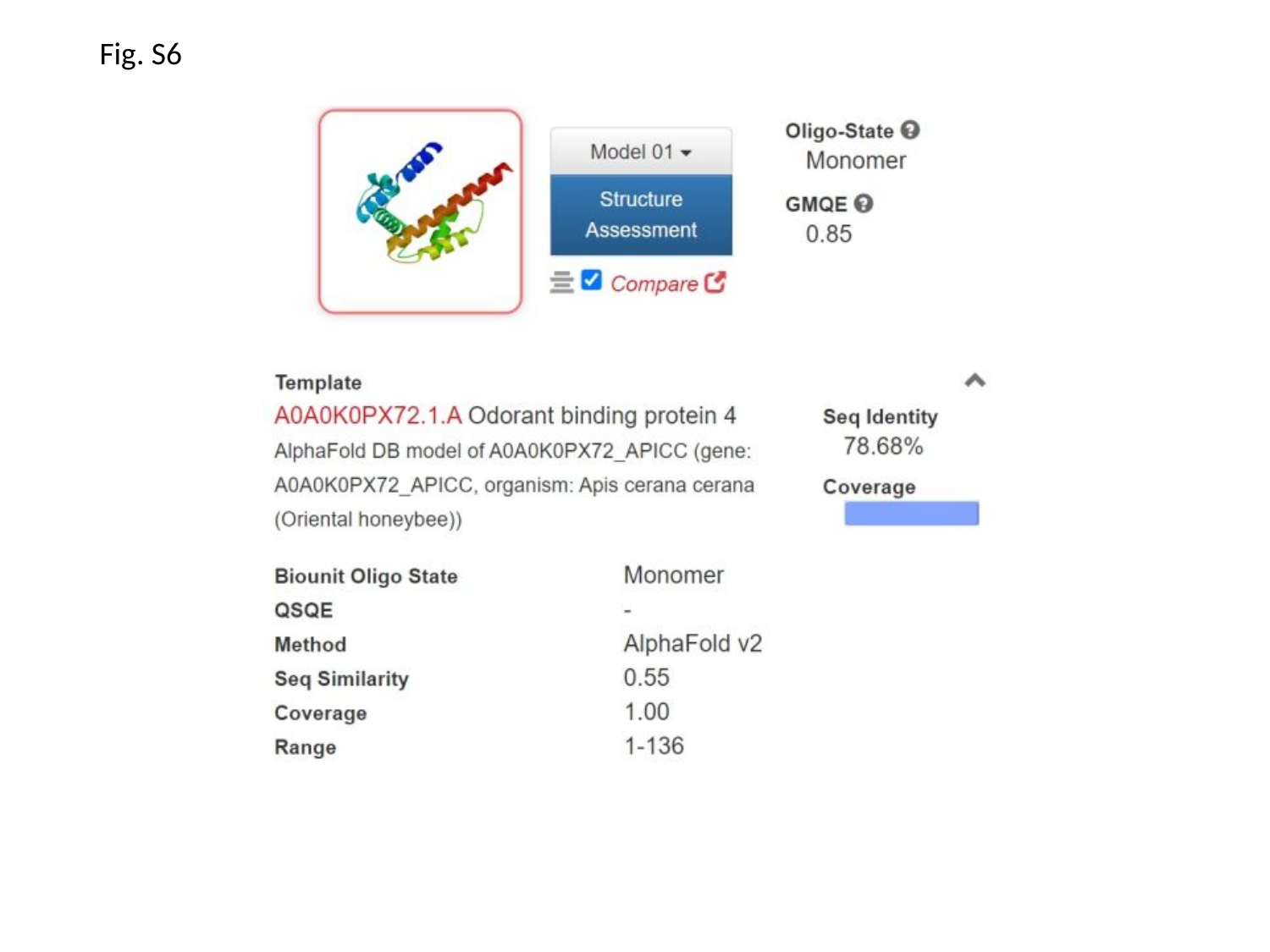

Fig. S6
